# Supplementary material for: Mechanisms governing the pioneering and redistribution capabilities of the non-classical pioneer PU.1
Source: Nat Commun. 2020 Jan 21;11:402. doi: 10.1038/s41467-019-13960-2 (PMC6972792; doi:10.1038/s41467-019-13960-2)
Supplement: Supplementary file 7 — Source data [file 41467_2019_13960_MOESM7_ESM.zip › Source_Data/Figure5/Figure5A_MotifScanOutput/homerResults/motif63.similar.html]

motif63

## Information for motif63

C
T
A
G
A
G
T
C
C
T
A
G
C
A
T
G
A
T
C
G
C
T
G
A
A
C
G
T
G
C
T
A
C
G
A
T
T
G
C
A
C
T
G
A
A
C
G
T
A
G
T
C
C
G
A
T
  
Reverse Opposite:  

C
G
T
A
T
C
A
G
T
G
C
A
A
G
C
T
A
C
G
T
G
C
T
A
C
G
A
T
T
G
C
A
A
G
C
T
T
A
G
C
G
T
A
C
G
A
T
C
T
C
A
G
A
G
T
C
  

|  |  |
| --- | --- |
| p-value: | 1e-25 |
| log p-value: | -5.760e+01 |
| Information Content per bp: | 1.675 |
| Number of Target Sequences with motif | 42.0 |
| Percentage of Target Sequences with motif | 1.39% |
| Number of Background Sequences with motif | 53.7 |
| Percentage of Background Sequences with motif | 0.12% |
| Average Position of motif in Targets | 193.4 +/- 131.9bp |
| Average Position of motif in Background | 206.7 +/- 164.4bp |
| Strand Bias (log2 ratio + to - strand density) | 0.4 |
| Multiplicity (# of sites on avg that occur together) | 1.00 |
| Motif File: | file (matrix) reverse opposite |

### Similar de novo motifs found

|  |  |  |  |  |  |  |  |
| --- | --- | --- | --- | --- | --- | --- | --- |
| Rank | Match Score | Redundant Motif | P-value | log P-value | % of Targets | % of Background | Motif file |
| 1 | 0.819 | A G T C T C G A C T A G A T C G G C T A C A G T C T G A C A G T C G T A C G T A A C G T T A G C | 1e-21 | -50.248159 | 1.26% | 0.12% | motif file (matrix) |
| 2 | 0.889 | C T A G G A T C C T A G C T A G A C T G C G T A A C T G C T G A C A G T T G C A C T G A C A G T A T G C | 1e-21 | -49.368313 | 1.29% | 0.13% | motif file (matrix) |
